# Properties and Perspectives of Rb_2_Co(SO_4_)_2_(H_2_O)_6_ Tutton Crystal: A Combined Experimental-Theoretical Analysis

**DOI:** 10.1021/acsomega.5c07896

**Published:** 2025-10-13

**Authors:** João G. de Oliveira Neto, Letícia F. Gomes, Francisco W. S. de Sousa Junior, Djany S. Silva, Kamila R. Abreu, Luiz F. L. da Silva, Luzeli M. da Silva, Pedro de F. Façanha Filho, Eliana B. Souto, Adenilson O. Dos Santos, Rossano Lang

**Affiliations:** † Center for Social Sciences, Health, and Technology, 37892 Federal University of Maranhão, 65900-410 Imperatriz, Maranhão, Brazil; ‡ Criminalistics Institute, Scientific Police of Pará, 68507-000 Marabá, Pará, Brazil; § UCD School of Chemical and Bioprocess Engineering, 8797 University College Dublin, Dublin, 4, Belfield D04 V1W8, Ireland; ∥ Institute of Science and Technology, 37871 Federal University of São Paulo, São José dos Campos, São Paulo 12231-280, Brazil

## Abstract

This paper presents a comprehensive investigation of a Tutton crystal, rubidium cobalt sulfate hexahydrate Rb_2_Co­(SO_4_)_2_(H_2_O)_6_, detailing its synthesis and characterizing its structural (PXRD), vibrational (FT-IR and Raman), thermal (TG and DSC), and optical (UV-vis-NIR) properties. Complementary, calculations using density functional theory (DFT) were implemented to estimate electronic band structure and assign optical phonon modes identified through FT-IR and Raman spectra. The material was prepared by the slow solvent evaporation method and crystallized, having *P*2_1_/*a*-space group in the monoclinic system (unit cell parameters *a* = 9.204(9) Å, *b* = 12.467(2) Å, *c* = 6.246(3) Å, β = 106.02(5)°, and *V* = 688.93(4) Å^3^). Hirshfeld surface analysis and void calculations revealed a densely packed structure stabilized by strong O···H/H···O hydrogen bonds, followed by O···Co/Co···O contacts, with a void volume of only 1.4%. Thermograms show a full dehydration at ≈ 384 K (Δ*H* = 301.15 kJ/mol). While electronic band structure indicates an electronic bandgap of 3.00 eV, dominated by Co^2+^ *d*-orbital contributions, the optical measurements display an optical bandgap of ≈ 4.13 eV, attributed to ligand-to-metal charge transfer bands involving electron donation from the nonbonding orbitals of H_2_O to the Co^2+^ orbitals. The optical absorbance (200–300 nm) transmittance (300–420 nm/580–1100 nm) windows underscore the potential of Rb_2_Co­(SO_4_)_2_(H_2_O)_6_ crystal.

## Graphical abstract

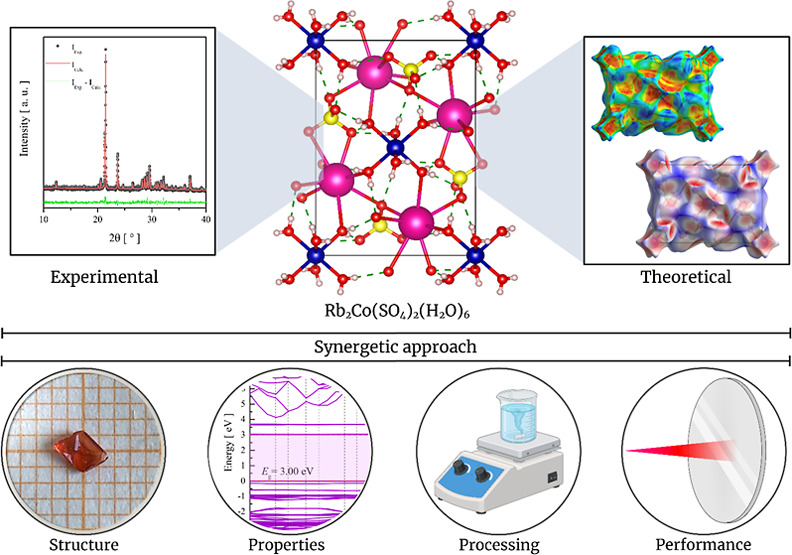

## 1 Introduction

Tutton salts constitute an important class of inorganic crystalline compounds named in honor of British chemist Alfred Edwin Howard Tutton, who pioneered their systematic study in the 19^th^ century.^1^ They belong to a family of hexahydrate double salts, characterized by the general formula M_2_M′(XO_4_)_2_(H_2_O)_6_.^2,3^ In this composition, M corresponds to monovalent cations such as NH_4_^+^, K^+^, Rb^+^, or Cs^+^; M′ represents divalent cations like Mg^2+^, V^2+^, Co^2+^, Ni^2+^, Cu^2+^, or Zn^2+^. High oxidation state elements, such as S^6+^ or Se^6+^, typically occupy X.^4,5^ These compounds crystallize in the monoclinic system of space group *P*2_1_/*a* or *P*2_1_/*c* (alternating the *a* and *c* lattice parameters in structural solution), forming prismatic solids with remarkable physicochemical properties.^6^

Among the various members of this family, Rb_2_Co­(SO_4_)_2_(H_2_O)_6_ stands out for its promising optical properties.^7,8^ The presence of Co^2+^ ions in an octahedral environment enables selective light absorption in the visible region, making it an attractive candidate for optical filter applications.^9–11^ The Tutton crystals like Rb_2_Co­(SO_4_)_2_(H_2_O)_6_ face challenges such as thermal instability and degradation at temperatures > 383 K, and these issues can be addressed through structural change (e.g., cations combination), coating (encapsulation) and even tailored synthesis approaches, which highlight the importance of a deeper understanding of their fundamental properties before moving on to modifications.^12^

The intrinsic interplay between chemical composition, crystal structure, and optical performance in Tutton salts remains poorly understood.^13,14^ And although the characteristic absorption bands of Co^2+^ ions in octahedral geometry are well documented,^15,16^ the effect of structural modifications, such as partial substitution of Rb^+^ with other alkali cations or controlled introduction of impurities, and spectral selectivity has not been sufficiently explored. This knowledge gap limits the design of optical filters with tunable spectral responses, which are essential for advanced applications such as polarized light sensing, along with selective wavelength blocking.^12,17,18^

In this scenario, computational chemistry emerges as an indispensable tool, offering deep insights into the electronic structure, spectroscopic properties, and thermal stability of a material.^19^ While conventional experimental methods face limitations in terms of cost, time, and spatial resolution, computational simulations allow systematic investigation of the structure-property relationships at the nanoscale.^20,21^ Density functional theory (DFT) has proven particularly valuable for these studies, enabling accurate prediction of electronic configurations and vibrational characteristics through first-principles calculations.^22,23^ The ability of DFT to model ground-state properties can clarify the role of the Co^2+^ coordination environment and its influence on optical behavior.^24^ Furthermore, molecular dynamics simulations complement DFT by helping understand thermal degradation mechanisms and structural stability under varying environmental conditions.^25,26^

Complementing these approaches, Hirshfeld surface analysis and the identification of voids (empty spaces) within the crystal structure provide valuable information about intermolecular interactions and atomic packing.^27–29^ Hirshfeld surfaces, obtained through the partitioning of the total electron density into atomic contributions, enable a precise mapping of contact regions between different structural components, including metal ions (Rb^+^ and Co^2+^), sulfate groups [SO_4_]^2–^, and coordinated H_2_O molecules.^30,31^ This analysis helps to elucidate how electronic polarization and charge-density redistribution affect selective light absorption,^32,33^ since the interactions between M′ ions and their ligands determine the observed *d*-*d* transitions in the ultraviolet-visible (UV-vis) spectrum.

Voids in the crystal structure play an equally crucial role in the stability and functionality of a material.^34^ In Rb_2_Co­(SO_4_)_2_(H_2_O)_6_, the presence of channels or cavities in the lattice directly influences H_2_O molecule diffusion under heating or desiccation conditions, affecting the robustness of optical filtration in different environments.^17^ Tools, such as CrystalExplorer, quantify the void volume percentage in the unit cell and predict how the loss of H_2_O or host molecule insertion may alter the structure and, consequently, the property of interest.^35^ The identification of voids then paves a way for material engineering strategies, where incorporation of organic or inorganic molecules can improve thermal stability without compromising optical translucency or spectral selectivity.^36–38^

Integrating advanced computational approaches with conventional experimental techniques comprises a tactic for addressing current challenges. A detailed analysis of Hirshfeld surfaces and voids can support a rational design of materials with enhanced stability and controlled optical performance.^35,39^ Such a convergent methodology would not only facilitate the advancement of Tutton crystals to practical applications in optical devices but also establish an exemplar for developing new functional materials based on the Tutton family salts.

Although some studies have investigated the optical behavior of Co^2+^-based Tutton salts,^1,9,40^ most have focused on isolated spectroscopic measurements without correlating them to detailed structural features such as voids or intermolecular interactions. Moreover, the combined use of experimental data, DFT simulations, and Hirshfeld surface analysis remains underexplored in this context.

In this work, structure-property relationships are investigated, primarily to elucidate the correlation between crystal packing and electronic structure and their impact on the optical response of Rb_2_Co­(SO_4_)_2_(H_2_O)_6_. For this purpose, single crystals were grown using the slow solvent evaporation method, followed by a comprehensive experimental study of their structural, thermal, vibrational, and optical properties. Hirshfeld surface analysis and void calculations provided an overview of the intermolecular interactions and lattice packing, while DFT calculations supported an accurate description of the normal vibration modes observed in experimental FT-IR and Raman spectra, as well as the characterization of the electronic band structure. The optical findings revealed several absorbance and transmittance bands in the range of 200 to 1100 nm, highlighting the potential of this material for application in light filtering devices. The experimental-theoretical approach used not only provides fundamental properties of this Tutton salt but also offers insight into the voids topology, electronic structure, as well as their optical behavior.

## 2 Experimental and Theoretical Procedures

### 2.1 Crystal Growth

Rb_2_Co­(SO_4_)_2_(H_2_O)_6_ salt, named as RbCoSOH (Rb = rubidium, Co = cobalt, S = sulfur, O = oxygen, H = hydrogen), was synthesized via slow solvent evaporation using equimolar amounts of Rb_2_SO_4_ and Co­(SO_4_)­(H_2_O)_7_ (both of 99% purity, purchased from Sigma-Aldrich, St. Louis, MO, USA) dissolved in 40 mL of deionized water under continuous magnetic stirring at 360 rpm for 180 min with a temperature kept at 313 K. The obtained solution was passed in a cellulose acetate filter paper of 25 μm cutoff size (Sigma-Aldrich, St. Louis, MO, USA), and then transferred to a beaker container with perforated polyvinyl chloride film (Sigma-Aldrich, St. Louis, MO, USA) to allow controlled evaporation, followed by storage in an oven at 308 K for solid-phase nucleation and crystal growth. Scheme 1 describes the chemical reaction involved in the product formation and the experimental synthesis procedure.

**Scheme 1:**
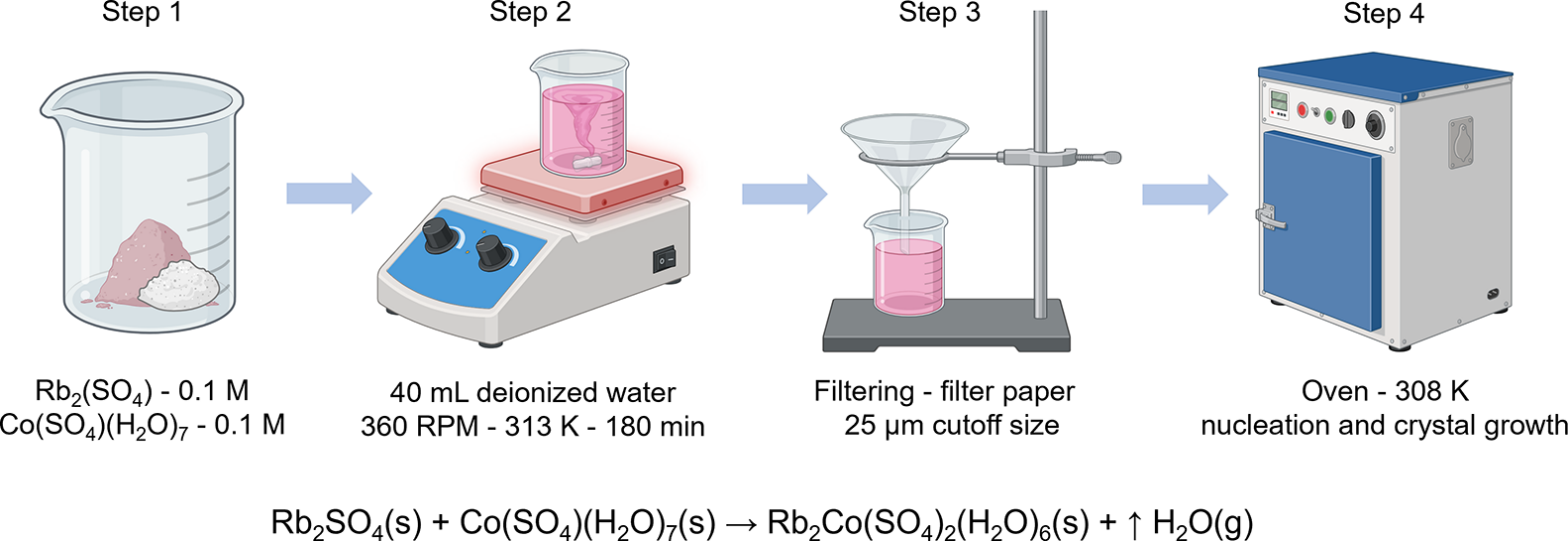
Experimental Procedure Used in the Synthesis of RbCoSOH Crystals

### 2.2 Experimental Characterization Techniques

The crystal structure was identified using powder X-ray diffraction (PXRD). The measurement was performed on a Panalytical Empyrean diffractometer (Malvern Panalytical, Malvern, UK) equipped with Cu Kα1 radiation (λ = 1.54056 Å), operating at 40 kV and 40 mA. Data were collected at room temperature, in the 2θ range of 10–40° with a step size of 0.02° and an acquisition time of 2 s per step. The experimental PXRD pattern was refined using the Rietveld method and the GSAS/EXPGUI software, with initial structural parameters derived from existing literature to confirm the crystalline phase.^7^

Fourier transform infrared (FT-IR) spectrum was obtained on a Bruker Vertex 70 V spectrophotometer (Billerica, MA, USA). The analysis involved the preparation of a pellet containing 2% of the powder crystal and 98% KBr (≥ 99% purity, Sigma-Aldrich, St. Louis, MO, USA). Spectrum was recorded in the mid-infrared range (4000–400 cm^–1^) with a resolution of 4 cm^–1^ and an average of 32 scans to improve the signal-to-noise ratio.

Raman spectroscopy was performed using a triple spectrometer model Trivista 557 (Princeton Instruments, Trenton, NJ, USA), operating in subtractive configuration, where only the last dispersion grating was applied. The system is coupled to a charge-coupled device (CCD) detector, the Pixis 256E, which is thermoelectrically cooled by the Peltier effect. The excitation source was a solid-state laser from the Cobolt brand, operating at a wavelength of 532 nm, with a power of 168 mW. The beam was focused on the sample using a Horiba Jobin Yvon microscope, model Olympus BX-41 (Horiba, Kyoto, Japan). Although this model allows for magnifications of up to 50×, a 50× objective lens was used in this study. The average laser power at the sample surface was ≈ 5.7 mW. The low laser power on the sample avoided any heating or dehydration effects. The measurement was performed in backscattering geometry, with four accumulations of 30 s each, covering the spectral range from 50 to 3900 cm^–1^, with an approximate spectral resolution of 2 cm^–1^.

Thermogravimetric and Differential Scanning Calorimetry (TG-DSC) analyses were conducted in a STA 449 F3 Jupiter thermal analyzer (Netzsch, Selb, Germany) equipped with an oven for simultaneous analysis of the sample and using an empty alumina crucible as reference. The heating rate was set to 10 K/min under a nitrogen atmosphere (flow rate of 100 mL/min), with a temperature range from 300 to 700 K.

For structural (PXRD), vibrational (FT-IR and Raman spectroscopy), and thermal (TG-DSC) analyses, crystals were ground in an agate mortar and pestle, and then sieved through a stainless-steel mesh filter with a 20 μm pore size (Thermo Fisher Scientific, Waltham, MA, USA) to ensure homogeneity.

UV-vis-NIR absorbance and transmittance spectra (200–1100 nm range) were obtained using a Thermo Evolution 220 double-beam spectrophotometer (Thermo Scientific, Waltham, MA, USA). This system, equipped with a deuterium excitation source, enabled the simultaneous measurement of both absorbance and transmittance spectra. For that, a suitable single crystal was selected and subjected to sequential surface polishing using abrasive papers with progressively finer grit sizes (240, 600, and 1200 mesh). The measurements were performed on the unoriented crystal and unpolarized light.

### 2.3 Computational Methods

Intermolecular interactions were computed using a method based on periodic theoretical approaches. The calculations were performed using CrystalExplorer 17 software,^35^ which enables accurate modeling of crystalline properties. Three-dimensional (3D) Hirshfeld surfaces were generated and analyzed using the normalized distance (*d*_norm_) contact, which accounts for both external (*d*_e_) and internal (*d*_i_) atomic positions relative to the surface, along with their respective van der Waals radii (*r*_vdW_). This procedure enables a detailed, qualitative, and quantitative characterization of the individual contributions of each noncovalent interaction.^41^ Additionally, void spaces within the unit cell were examined using procrystal electron density isosurfaces (set at 0.002 au).^28^

The electronic and vibrational properties of RbCoSOH were investigated using DFT-periodic calculations as implemented in the Cambridge Serial Total Energy Package (CASTEP).^42^ Norm-conserving pseudopotentials were employed to represent the core electrons,^43^ while the exchange-correlation effects were treated within the Generalized Gradient Approximation (GGA) using the Perdew-Burke-Ernzerhof (PBE) functional.^44^ Brillouin zone integration was performed using a 2 × 2 × 2 Monkhorst-Pack *k*-point mesh. A high energy cutoff of 820 eV was used for the plane-wave basis set, ensuring well-converged results. The atomic positions were optimized using the Broyden-Fletcher-Goldfarb-Shanno (BFGS) algorithm until the following convergence criteria were met: a maximum energy change of 1.0 × 10^–6^ eV/atom, a maximum force of 0.03 eV/Å, a maximum stress of 0.1 GPa, and a maximum displacement of 0.001 Å.^45^ DFT method was used by coupling the Hubbard U correction (DFT + U) to account for the *d*-orbital electron correlation effects in the Co atom.^46^ Following structural optimization, the electronic band structure and related properties were calculated by propagating the electronic wave function along high-symmetry points in the Brillouin zone. The chosen *k*-point path was Z­(0.000, 0.000, 0.500); Γ­(0.000, 0.000, 0.000); Y­(0.000, 0.500, 0.000); A­(−0.500, 0.500, 0.000); B­(−0.500, 0.000, 0.000); D­(−0.500, 0.000, 0.500); E­(−0.500, 0.500, 0.500); C­(0.000, 0.500, 0.500). The calculations were performed on a monoclinic cell (*P*2_1_/*a*-space group) containing a total of 62 atoms.

## 3 Results and Discussion

### 3.1 Synthesis of the Crystal and Structural Characterization

The inset in Figure 1a displays a RbCoSOH single crystal with a prismatic morphology and deep red coloration, characteristic of *d-d* electronic transitions originating from Co^2+^ ions in an octahedral coordination environment.^16^ The sample was obtained after 21 days of crystallization in an acidic medium (pH ≈ 4.9), with average dimensions of 3.3 × 2.5 × 0.5 mm^3^ (L × W × H). The synthesis performed via slow solvent evaporation at 308 K yielded approximately 62.5% of the crystalline product.

**Figure 1:**
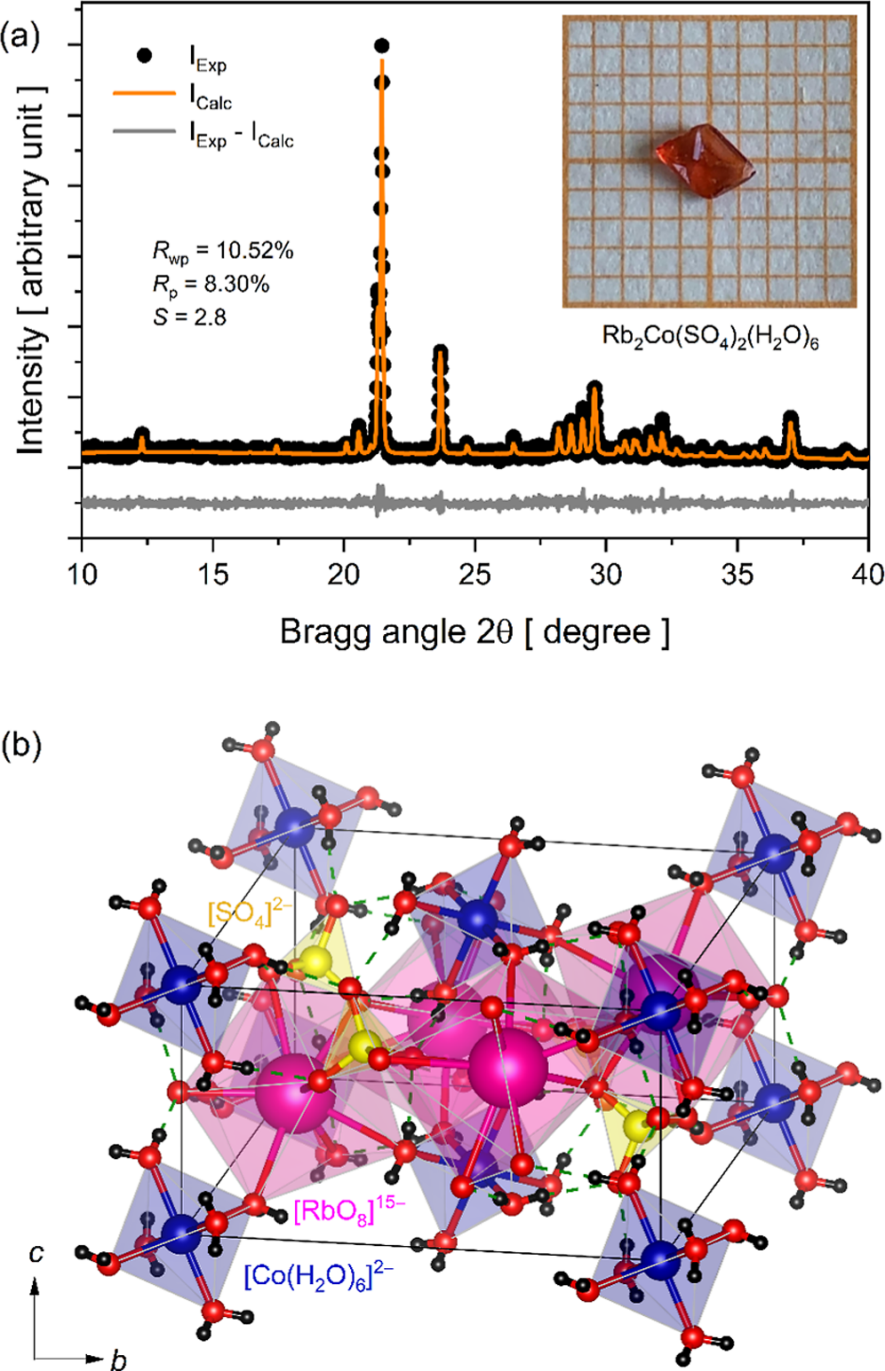
(a) Experimental PXRD pattern refined by the Rietveld method for the powdered RbCoSOH crystals. Inset: Image of an RbCoSOH single crystal grown by slow solvent evaporation. (b) Polyhedral representation of the primitive unit cell for the RbCoSOH Tutton salt.

The experimental PXRD pattern obtained from powdered crystals was refined using the Rietveld method to identify the structural phase and determine crystallographic parameters. For that, the crystallographic information file (CIF) number 409494 was used as a reference for structure comparison. The experimental and calculated diffractograms, along with the difference between them, are presented in Figure 1a. The results indicate that RbCoSOH crystallizes in the monoclinic system, *P*2_1_/*a*-space group, containing two formula Rb_2_Co­(SO_4_)_2_(H_2_O)_6_ per unit cell (*Z* = 2). The refined unit cell parameters were *a* = 9.204(9) Å, *b* = 12.467(2) Å, *c* = 6.246(3) Å, α = γ = 90°, β = 106.02(5)°, and *V* = 688.93(4) Å^3^. The refinement quality indicators (*R*_wp_ = 10.52%, *R*_p_ = 8.30%, and *S* = 2.8) demonstrate good agreement between the experimental phase and the theoretically reported parameters in the literature.^7^ These structural parameters confirm that the RbCoSOH crystal belongs to the isomorphous crystallographic family of rubidium Tutton salts.

Figure 1b displays a projection of the RbCoSOH unit cell along the *a*, *b*, and *c* axes, illustrated using coordination polyhedra. At the corners of the primitive cell, Co^2+^ ions are observed, coordinated by six H_2_O molecules in a distorted octahedral geometry due to the Jahn-Teller effect, a phenomenon typical of *d* ^7^ ions. These ions form [Co­(H_2_O)_6_]^2+^ hexaaqua complexes, which interact with the neighbors via hydrogen bonding and electrostatic interactions. The tetrahedral [SO_4_]^2–^ groups act as bridges between cobalt complexes, connecting them through O–H···O–S bonds. The Rb^+^ cations occupy sites between the [Co­(H_2_O)_6_]^2+^ octahedra and [SO_4_]^2–^ tetrahedra, forming [RbO_8_]^15–^ coordination polyhedra. These polyhedra establish a type of ionic channel, stabilized by interactions with oxygen from sulfate and H_2_O molecules. The resulting 3D framework exhibits alternating layers of [Co­(H_2_O)_6_]^2+^ octahedra and [SO_4_]^2–^ tetrahedra, with Rb^+^ ions acting as spacer species that maintain structural stability.

The monoclinic symmetry (β ≠ 90°) of RbCoSOH and the layered arrangement of [Co­(H_2_O)_6_]^2+^ octahedra and [SO_4_]^2–^ tetrahedra induce an anisotropic packing, which directly influences the directional propagation of light through the crystal. Such an anisotropy is reflected in the spectroscopic properties arising from Co^2+^ ions, as the orientation of the coordination polyhedra affects the polarization and absorption of incident light.

### 3.2 Study of Intermolecular Interactions from Hirshfeld Surfaces and Crystal Voids

Hirshfeld surface analysis was performed to complement the structural data and to provide a detailed characterization of the intermolecular interactions in RbCoSOH Tutton salt. Figure 2a depicts the refined unit cell with lattice parameters used for the electron density surface calculations. Figure 2b shows the plot of the Hirshfeld surface in terms of *d*_norm_ applying a three-color scheme that maps interaction strengths relative to van der Waals radii (*r*_vdW_): (i) red regions (distances < *r*_vdW_) reveal strong O···H/H···O hydrogen bonds predominantly around oxygen atoms, along with significant O···Co/Co···O coordination bonds and Rb···O/O···Rb electrostatic interactions; (ii) white areas (distances ≈ *r*_vdW_) indicate neutral contacts, and (iii) blue zones (distances > *r*_vdW_) correspond to weak or noninteracting surfaces. The concentration of red surfaces near O, H, Co, and Rb sites confirms that oxygen plays a dual role, i.e., as a hydrogen bond acceptor and as a metal coordination center, with the most intense interactions occurring between H_2_O molecules and sulfate groups, consistent with characteristic Tutton salt packing patterns.

**Figure 2:**
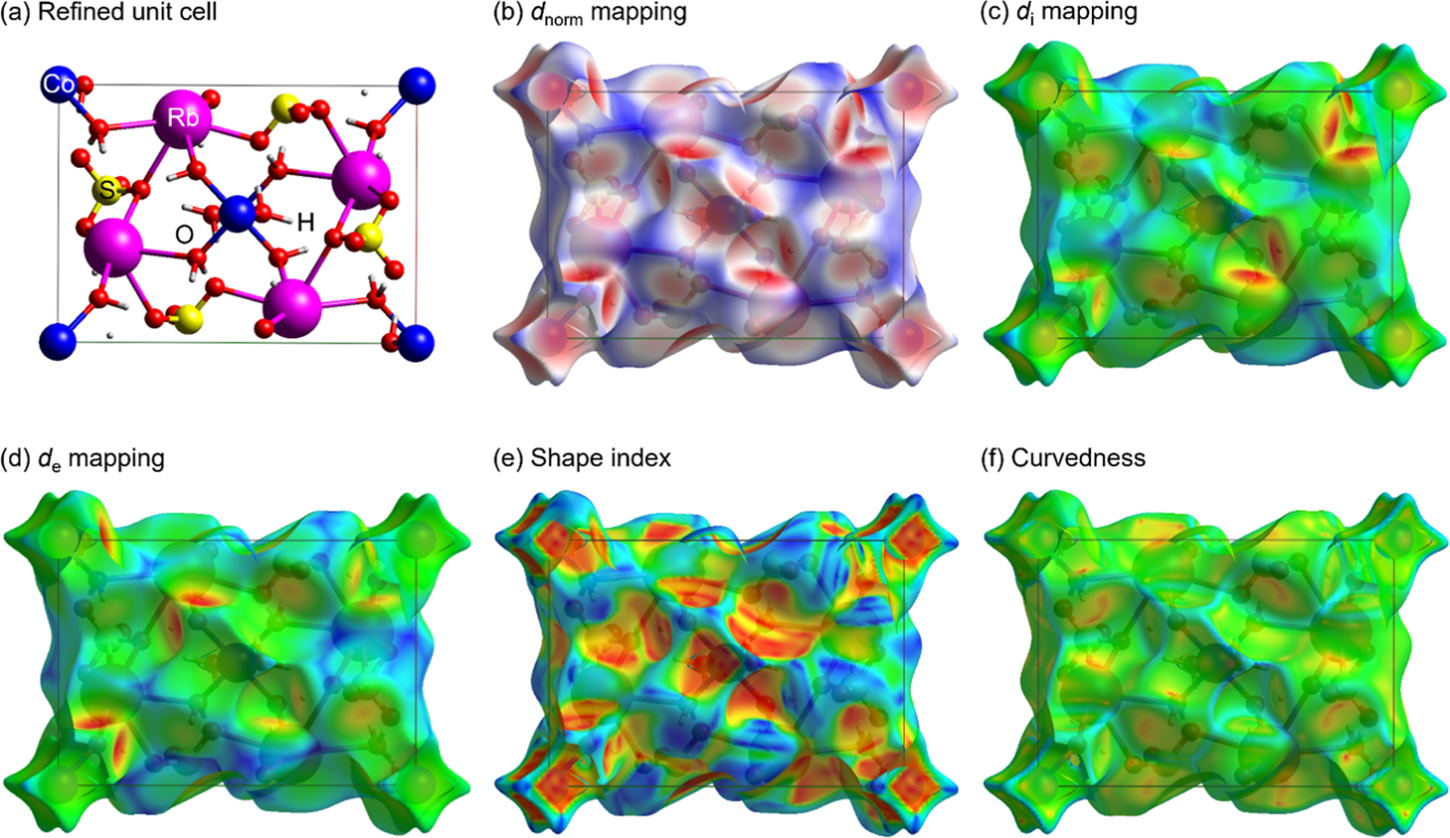
(a) Rietveld method-refined primitive unit cell of RbCoSOH Tutton salt. 3D Hirshfeld surfaces mapped with different molecular interaction properties: (b) *d*_norm_, (c) *d*_i_, (d) *d*_e_, (e) shape index, and (f) curvedness.

The surfaces shown in Figure 2c,d are complementary and map the *d*_i_ and *d*_e_ functions, respectively. The red regions in Figure 2c around the [Co­(H_2_O)_6_]^2+^ and [RbO_8_]^15–^ units correspond to donor sites for intermolecular interactions, while the warm-toned areas (yellow, orange, and red) near the [RbO_8_]^15–^ and [SO_4_]^2–^ layers in Figure 2d characterize acceptor sites. Together, these surfaces provide key insights into how molecular fragments interact with their neighborhood through propagation of the unit cell in real space.

The topology of intermolecular contacts in the crystal was further investigated through shape index and curvedness-mapped Hirshfeld surfaces.^47^ In Figure 2e, warm-colored regions surrounding the ionic units represent concave areas (negative curvature), indicating where neighboring layers interlock through inward-bending surfaces. Conversely, cool-colored zones correspond to convex features (positive curvature), demonstrating outward-protruding molecular stacking.^33^ The red areas highlight short-range, strong interactions (O–H···O hydrogen bonds and coordination contacts), while blue regions map long-range, weak van der Waals forces. Notably, the blue contours in Figure 2f delineate zones of maximum interaction density, revealing the most intense intermolecular contacts between ionic fragments, particularly at the [Co­(H_2_O)_6_]^2+^–[SO_4_]^2–^ and [Co­(H_2_O)_6_]^2+^–[RbO_8_]^15–^ interfaces.

A quantitative analysis of intermolecular interactions, as represented by 2D fingerprint plots, is shown in Figure 3. The cumulative histogram (100%) characterizes all contacts within the RbCoSOH unit cell. Through detailed deconvolution of each contribution, the interaction patterns showed to be dominated by three contact types: O···H/H···O (36.4%), O···Co/Co···O (27.9%), and Rb···O/O···Rb (11.6%). These specific interactions act as the primary stabilizing forces, providing long-range periodicity to the molecular layers and ensuring crystal packing through the formation of a stable lattice.

**Figure 3:**
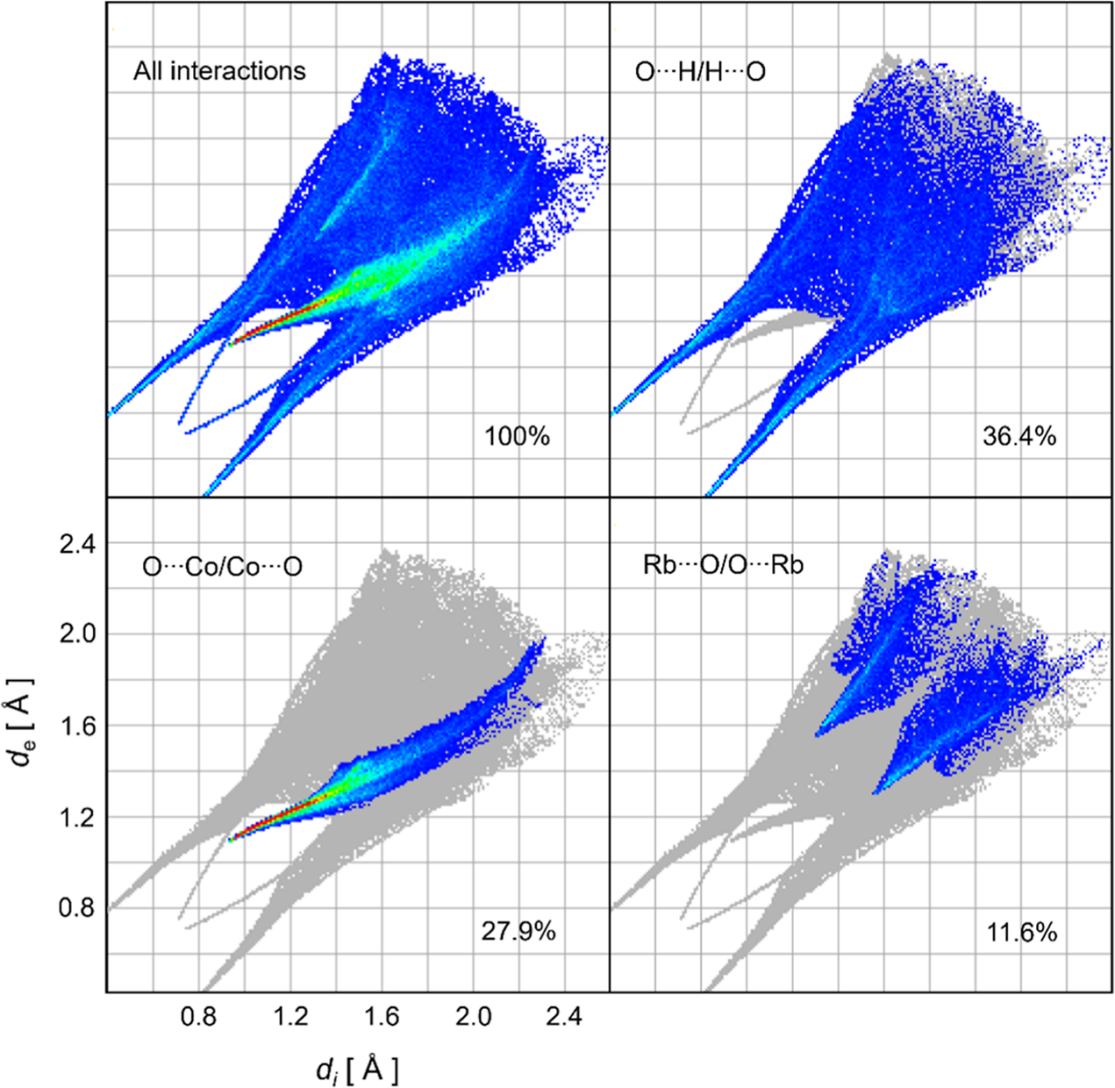
2D fingerprint plots (full and deconvoluted) generated from the refined unit cell of RbCoSOH Tutton salt.

Notably, while the O···H/H···O contacts represent the most abundant interactions (36.4%), their sharp peaks, particularly in the low *d*_e_ and *d*_i_ regions show high interaction intensity between molecular fragments, consistent with Tutton salt structures previously reported.^14,18,31^ The pronounced red spots for O···Co/Co···O contacts (27.9%) similarly indicate strong coordination bonds. Although the O···H/H···O, O···Co/Co···O, and Rb···O/O···Rb dominate the frame of intermolecular interactions, the quantitative analysis revealed secondary contacts: H···H (8.7%), O···O (5.5%), Co···H/H···Co (3.8%), S···O/O···S (3.8%), Rb···H/H···Rb (2.2%), and S···H/H···S (0.1%), which collectively stabilize the crystal.

Additionally, the crystal was investigated with a focus on its void characteristics, which play a crucial role in understanding the structural stability, hydration/dehydration behavior, and potential guest inclusion properties in the salt. As shown in Figure 4, the voids analysis indicates a low empty volume of 9.78 Å^3^, corresponding to 1.41% of the unit cell volume, demonstrating a densely packed structure. The low percentage of voids in the structure suggests limited free space for impurity and dopant introduction,^12^ consistent with the rigid framework formed by the large Rb^+^ cations, [SO_4_]^2–^ tetrahedra, and hydrogen-bonded H_2_O molecules. The surface area of the voids was calculated to be 57.2 Å^2^, reflecting the relatively confined nature of the crystal. However, the strategy of mixing monovalent cations smaller than Rb should increase these numbers.

**Figure 4:**
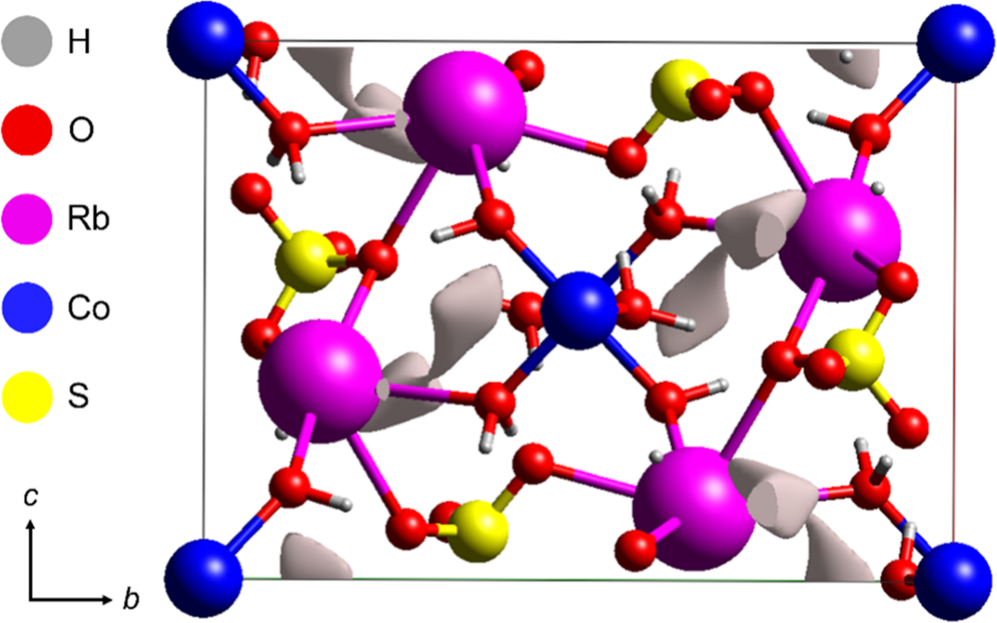
Crystal voids within the RbCoSOH primitive unit cell are visualized through isosurfaces along the *c*–*a* plane.

Furthermore, the globularity (0.387) and asphericity (0.137) parameters highlight the irregular, nonspherical morphology of the voids, which arise from the anisotropic arrangement of structural units and the asymmetric hydrogen-bonding lattice.^48^ These findings contribute to the broader understanding of Tutton crystals, where subtle variations in the void framework impact several properties such as thermal, vibrational, electronic, and optical. To the best of our knowledge, these computational methods (2D fingerprint plots, Hirshfeld surfaces, and voids analyses) have not been previously reported for rubidium-based Tutton salts, thereby contributing to a deeper structural characterization of this underexplored system.

### 3.3 Theoretical Studies via DFT

Using periodic DFT calculations, the primitive unit cell of RbCoSOH was successfully optimized, considering its propagation in the reciprocal lattice and the intermolecular interactions involved. Table 1 presents the relaxed cell dimensions compared to the experimental data obtained in this study via Rietveld refinement. All lattice parameters showed good agreement with each other, indicating that the choice of the GGA-PBE functional is suitable for analyzing the structural, electronic, and spectroscopic parameters of the rubidium-based Tutton salt.

**Table 1:** Relaxed Lattice Parameters Computed from the DFT Method and Compared with the Experimental Lattice Parameters from the Literature,^7^ and Calculated from the Rietveld Method for the RbCoSOH Tutton Salt

structural parameters	literature	Rietveld	DFT
*a* [Å]	9.197(2)	9.204(9)	9.229(3)
*b* [Å]	12.446(2)	12.467(2)	12.581(7)
*c* [Å]	6.236(10)	6.246(3)	6.320(3)
β [°]	106.04(10)	106.02(5)	105.55(7)

The electronic properties were described in terms of the band structure combined with orbital contributions through the PDOS (projected density of states), as shown in Figure 5. Figure 5a,b illustrate the band structure plots for the spin-up and spin-down channels, respectively. Both functions are represented by high-symmetry points, which designate the boundaries of the Brillouin zones, labeled as Γ, Y, A, B, D, and E. The plots display two distinct sets of bands, corresponding to the overlap of atomic orbitals that constitute the RbCoSOH structure: (i) last flat valence bands (underlying 0 eV) and (ii) first flat conduction bands (above 0 eV). For the spin-up channel at the Γ point, an electronic bandgap of 1.9 eV was recorded. In contrast, for the spin-down channel, there was a positive shift of 1.1 eV, resulting in a wide bandgap of 3.00 eV—considered the effective electronic bandgap of the RbCoSOH crystal.

**Figure 5:**
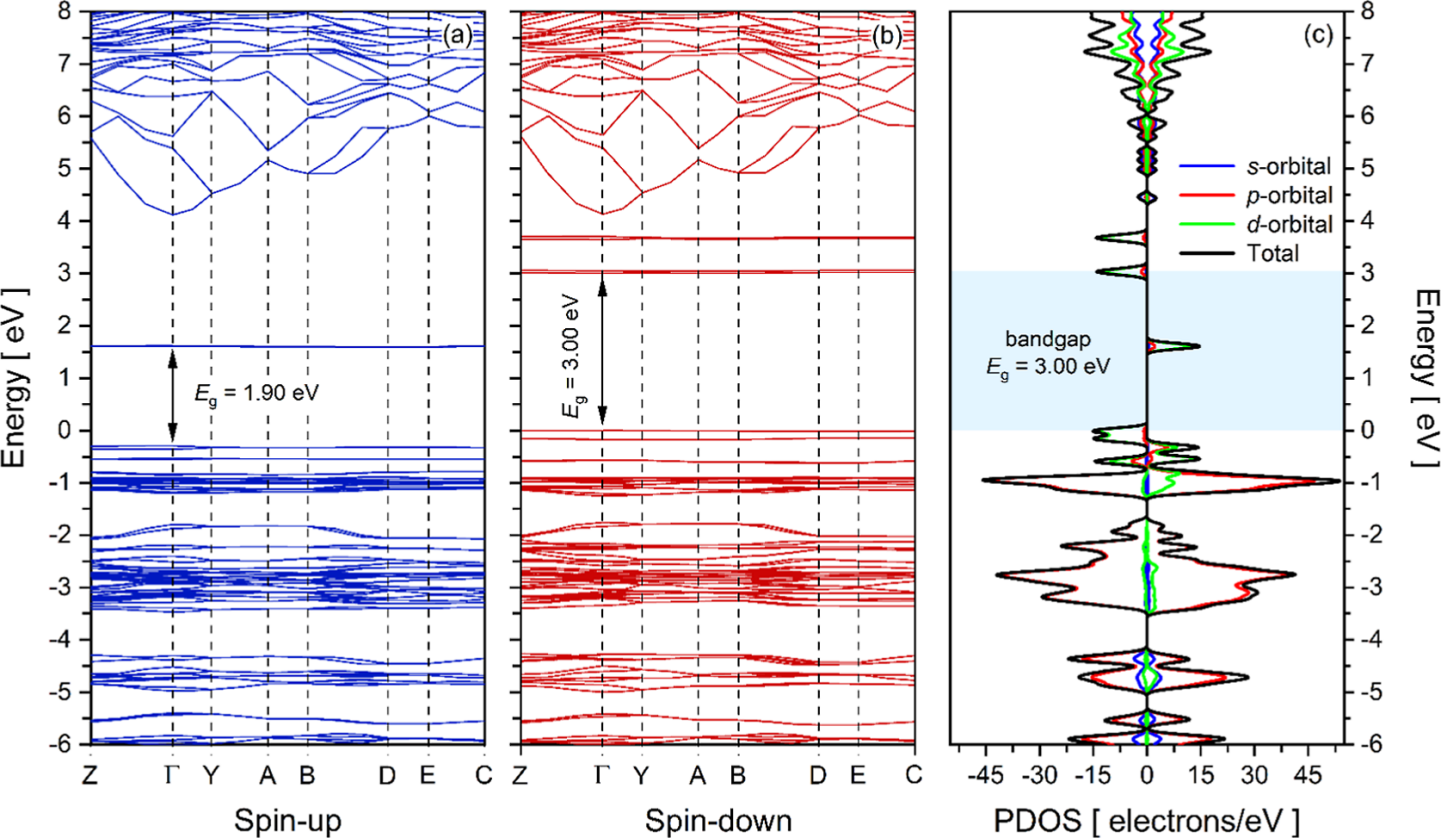
Band structure plots as a function of energy for: (a) spin-up and (b) spin-down states of the RbCoSOH crystal calculated via DFT. (c) PDOS computations by orbital contributions (*s*, *p*, and *d*). The Fermi level is set to zero in all plots.

Figure 5c depicts the PDOS total contribution. The PDOS deconvolution into contributions from specific atoms and orbitals, offering a detailed perspective on how electrons are distributed across different energy levels, is shown in Figure S1 (Supporting Information). Oxygen atoms dominate the valence band region, primarily through their 2*p*-orbitals, which form the upper edge of the valence band. Sulfur atoms exhibit a similar behavior, contributing to the valence band with minor participation in the conduction band. Cobalt atoms play a crucial role in both the valence and conduction bands, with strong 3*d*-orbital contributions near the Fermi level, particularly influencing the conduction band minimum. However, the lack of dispersion observed indicates that the Co 3*d* orbitals do not significantly hybridize with any other orbitals. Rubidium atoms (3*d*-orbitals) contribute mainly to states above 5 eV, confirming their ionic character within the structure. Hydrogen atoms (1*s*) show only minor contributions on both sides of the Fermi level, suggesting a limited impact on the electronic properties. These findings indicate that the bandgap is primarily governed by the interaction between 2*p* states (O and S) in the valence band and Co 3*d* states in the conduction band.

Generally, Tutton salts containing K^+^ or NH_4_^+^ monovalent cations in their chemical composition exhibit electronic bandgaps greater than 4.0 eV, characteristic of electrical insulating materials. K_2_Zn­(SO_4_)_2_(H_2_O)_6_ (4.66 eV),^49^ K_2_Mn_0.15_Co_0.85_(SO_4_)_2_(H_2_O)_6_ (4.13 eV),^22^ (NH_4_)_2_Fe­(SO_4_)_2_(H_2_O)_6_ (4.61 eV),^12^ and (NH_4_)_2_Zn­(SO_4_)_2_(H_2_O)_6_ (4.82 eV)^31^ are good examples. Therefore, the presence of Rb^+^ ions in the monovalent sites induces a significant distortion in the *d*- and *p*-state densities, leading to a narrowing of the bandgap to 3.00 eV. A similar phenomenon was observed in the Tutton salt (NH_4_)_2_Fe_0.11_Ni_0.89_(SO_4_)_2_(H_2_O)_6_,^18^ although with a higher bandgap value (3.99 eV), suggesting that the nature of the divalent cation also impacts the electronic properties of a Tutton crystal. It should be noted, however, that the calculated band gap is highly dependent on the specific exchange-correlation functional and computational parameters chosen for the DFT simulation.

### 3.4 Group Theory and Vibrational Characterization

As discussed in Section 3.1, RbCoSOH consists of three molecular layers in the primitive unit cell: Rb^+^, [Co­(H_2_O)_6_]^2+^, and [SO_4_]^2–^, containing 2, 19, and 10 atoms, respectively, based on the chemical formula Rb_2_Co­(SO_4_)_2_(H_2_O)_6_ (totaling 31 atoms per formula unit). Thus, the crystal contains 62 atoms per unit cell due to the presence of two formula units per cell (*Z* = 2). According to group theory for the *C*_2h_^5^-factor group and using the crystallographic data,^50^ this Tutton salt exhibits 186 degrees of freedom, which can be deconvoluted into irreducible representations: Γ^total^ = 45A_g_ + 48A_u_ + 45B_g_ + 48B_u_. Among these representations, A_g_ and B_g_ stand for Raman activity, while A_u_ and B_u_ characterize IR activity. However, there are three acoustic modes included in the total representation, which reduce to Γ^Raman^ = 45A_g_ + 45B_g_ and Γ^IR^ = 47A_u_ + 46B_u_. All the computed modes are provided in Table S1 (Supporting Information). Table 2 lists the observed vibration modes (both IR- and Raman-active) along with their calculated counterparts, irreducible representations, and respective assignments.

**Table 2:** Vibration Mode Analyses for the RbCoSOH Crystal: ω_Raman_ = Experimental Raman Modes, ω_IR_ = Experimental IR Modes, ω_Calc_ = Calculated Wavenumbers at Zero K, Irrep. = Irreducible Representation, and Their Assignments

ω_Raman_ [cm^–1^]	ω_IR_ [cm^–1^]	ω_Calc_ [cm^–1^]	Irrep.	assignments^a^
a-68		141	A_g_	trans[Rb_2_] + τ[Co(H_2_O)_6_] + δ[SO_4_]
b-89		155	A_g_	trans_op_[Rb_2_] + τ[Co(H_2_O)_6_] + δ[SO_4_]
c-108		162	B_g_	trans_op_[Rb_2_] + τ[Co(H_2_O)_6_] + δ[SO_4_]
d-122		179	A_g_	trans[Rb_2_] + δ[Co(H_2_O)_6_] + δ[SO_4_]
e-150		205	A_g_	δ[Co(H_2_O)_6_] + δ[SO_4_]
f-179		227	B_g_	δ[Co(H_2_O)_6_] + δ[SO_4_]
g-234		253	A_g_	δ[Co(H_2_O)_6_] + δ_s_[SO_4_]
h-271		276/286	B_g_/A_g_	δ_s_[Co(H_2_O)_6_] + δ_s_[SO_4_]
i-306		300/318	A_g_	δ[Co(H_2_O)_6_]
j-401		411	B_g_	wag[H_2_O] + δ_s_[SO_4_]
	406	424	B_u_	ν_as_[Co(H_2_O)_6_] + ν_s_[SO_4_]
	433	441	B_u_	ν_s_[Co(H_2_O)_6_] + ν_s_[SO_4_]
	444	459	B_u_	ν_as_[Co(H2O)6] + ν_s_[SO_4_]
	459	469	A_u_	wag[H_2_O] + δ_s_[SO_4_]
k-463		438	A_g_	wag[H_2_O] + δ_s_[SO_4_]
l-470		455	A_g_	wag[H_2_O] + δ_s_[SO_4_]
	501	576	B_u_	wag[H_2_O]
	520	596	B_u_	wag[H_2_O]
	541	607	A_u_	wag[H_2_O] + δ_as_[SO_4_]
	566	658	B_u_	tw[H_2_O]
	583	682	A_u_	tw[H_2_O]
	612	706	A_u_	tw[H_2_O]
m-622		586	B_g_	wag[H_2_O]
	632	716	B_u_	tw[H_2_O]
n-643		613	A_g_	wag[H_2_O] + δ_as_[SO_4_]
	738	797	B_u_	ρ[H_2_O]
	758	805	A_u_	ρ[H_2_O]
o-785		702/715	A_g_	tw[H_2_O]
	788	845	B_u_	ρ[H_2_O]
	813	871	B_u_	ρ[H_2_O]
	849	905	A_u_	ρ[H_2_O]
p-867		844/857	A_g_	ρ[H_2_O]
	872	912	B_u_	ρ[H_2_O] + ν_s_[SO_4_]
	895	937	B_u_	ρ[H_2_O] + ν_s_[SO_4_]
	945	944	B_u_	ρ[H_2_O] + ν_s_[SO_4_]
	983	964	A_u_	wag[H_2_O] + ν_s_[SO_4_]
q-999		969	B_g_	wag[H_2_O] + ν_as_[SO_4_]
	1087	1061	B_u_	wag[H_2_O] + ν_as_[SO_4_]
r-1094		1053/1068	A_g_	tw[H_2_O] + ν_as_[SO_4_]
	1099	1080	B_u_	tw[H_2_O] + ν_as_[SO_4_]
	1114	1086	A_u_	wag[H_2_O] + ν_as_[SO_4_]
s-1122		1095	B_g_	wag[H_2_O] + ν_as_[SO_4_]
	1138	1119	B_u_	wag[H_2_O] + ν_as_[SO_4_]
t-1139		1122	A_g_	wag[H_2_O] + ν_as_[SO_4_] + ν_s_[SO_4_]
u-1168		1054	B_g_	wag[H_2_O] + ν_as_[SO_4_] + ν_s_[SO_4_]
	1566	1558	A_u_	wag[H_2_O] + ν_as_[SO_4_]
	1690	1586/1598	A_u_/B_u_	wag[H_2_O] + ν_as_[SO_4_]
	3141	3040	B_u_	ν_s_[H_2_O]
v-3210		3113	B_g_	ν_as_[H_2_O]
	3222	3066	A_u_	ν_s_[H_2_O]
	3284	3137	B_u_	ν_s_[H_2_O]
x-3330		3244	B_g_	ν_as_[H_2_O]
	3421	3213	B_u_	ν_as_[H_2_O]

a Trans = translational; trans_op_ = translational out-of-phase; τ = torsion; tw = twisting; wag = wagging; ρ = rocking; δ = bending; δ_a_ = antisymmetric bending; δ_s_ = symmetric bending; ν_a_ = antisymmetric stretching; ν_s_ = symmetric stretching.

Upon close inspection, considerable shifts (up to Δν ≈ 100 cm^–1^) in some calculated modes are observed. This is because the calculations are performed for a crystal at absolute zero (0 K). The experiments, however, were conducted at room temperature. At this temperature, the material expands, and molecules occupy higher vibrational energy levels. The thermal energy causes peak broadening and a general shift compared to the 0 K theoretical model. Anharmonicity effects must also be considered.

#### 3.4.1 FT-IR Spectroscopy

Figure 6 shows the experimental FT-IR spectrum of the powdered crystal in the spectral range of 4000 to 400 cm^–1^. In the high-frequency region, a broad absorption band is observed between 3800 and 2700 cm^–1^, corresponding to the symmetric and antisymmetric stretching modes of H_2_O molecules coordinated to the metal center. According to the literature,^51^ this broadband occurs due to the high polarity of water in the IR region. Additionally, DFT calculations suggest the presence of 12 vibration modes overlapping in this wavelength region, associated with the motions of the six H_2_O units.^12,31^ These modes have also been observed in Rb_2_Mg­(SO_4_)_2_(H_2_O)_6_ crystals doped with VO^2+^ and Cu^2+^.^52,53^

**Figure 6:**
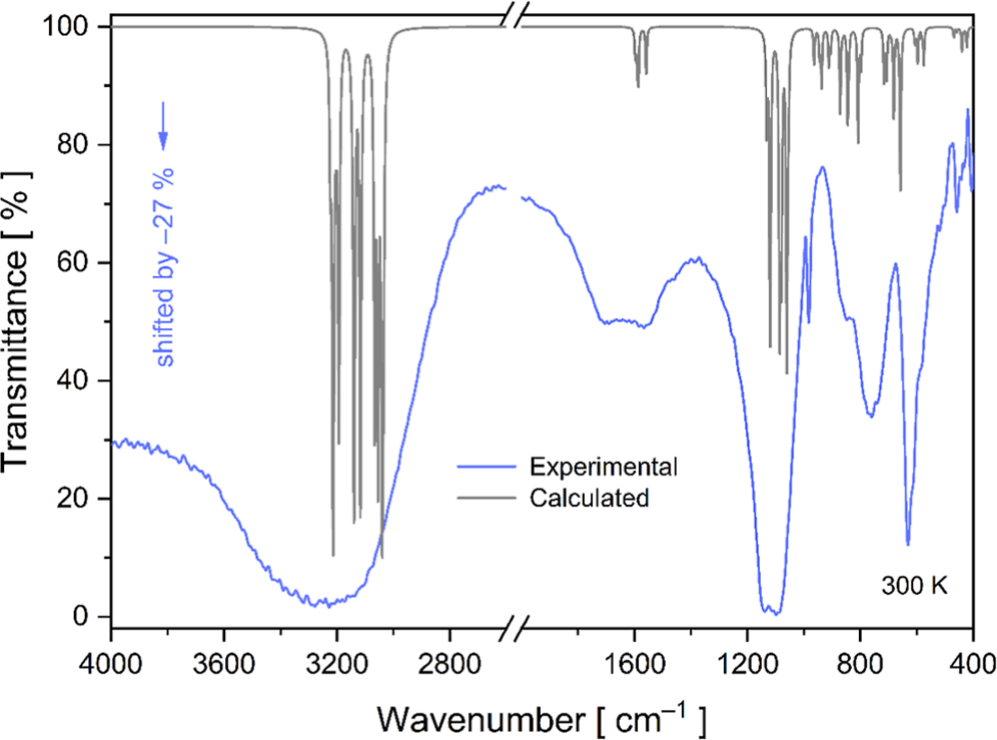
Experimental and calculated FT-IR spectra of the powdered RbCoSOH Tutton crystal.

In the spectral range of 1700 to 460 cm^–1^, twenty-three IR vibrational modes were observed, associated with several types of H_2_O vibrations (wagging, twisting, and rocking), with minor contributions from coupled motion of the [SO_4_]^2–^ tetrahedra (symmetric stretching, asymmetric stretching, asymmetric bending, and symmetric bending). The previously discussed Hirshfeld surface data (Section 3.2) support the results, revealing that the RbCoSOH crystal is primarily stabilized by strong hydrogen bonds between the [SO_4_]^2–^ and [Co­(H_2_O)_6_]^2+^ ions.

In the spectral region below 460 cm^–1^, three weak-intensity absorption bands were detected at ≈ 444, 433, and 406 cm^–1^. These bands were properly assigned to symmetric and asymmetric stretching vibrations of the [Co­(H_2_O)_6_]^2+^ hexahydrate complex, with contributions from symmetric stretching modes of the [SO_4_]^2–^ tetrahedra. Under *C*_2h_^5^-factor group, [SO_4_]^2–^ and [Co­(H_2_O)_6_]^2+^ assume symmetry lower than *T*_*d*_ (*C*_1_ site symmetry) and *O*_*h*_ (*C*_i_ site symmetry), respectively.^3,40,54^ In addition, Tutton salts tend to exhibit low-intensity bands in the lower frequency region, associated with hexaqua-complexes, due to the high molecular weight of the metals and the strong covalent bonds within the coordination compound.^55,56^

#### 3.4.2 Raman Spectroscopy

Figure 7a shows the unpolarized Raman spectrum of a powdered crystal in the spectral range of 40 to 3800 cm^–1^. Consistent with the IR modes, a broad and intense band is observed in the high wavenumber region (2800–3800 cm^–1^), corresponding to the antisymmetric and symmetric stretching vibrations of H_2_O molecules. This broadband indicates an extensive hydrogen bonding lattice within the crystal lattice. These interactions are crucial for maintaining structural stability and lattice periodicity. According to Oliveira Neto et al.,^57^ 12 fingerprint Raman modes of H_2_O molecules are present beneath this band in Tutton salts. Furthermore, several other isolated H_2_O vibration modes are recorded at lower wavelengths, including a rocking mode at 867 cm^–1^, a twisting mode at 785 cm^–1^, and a wagging mode at 622 cm^–1^.

**Figure 7:**
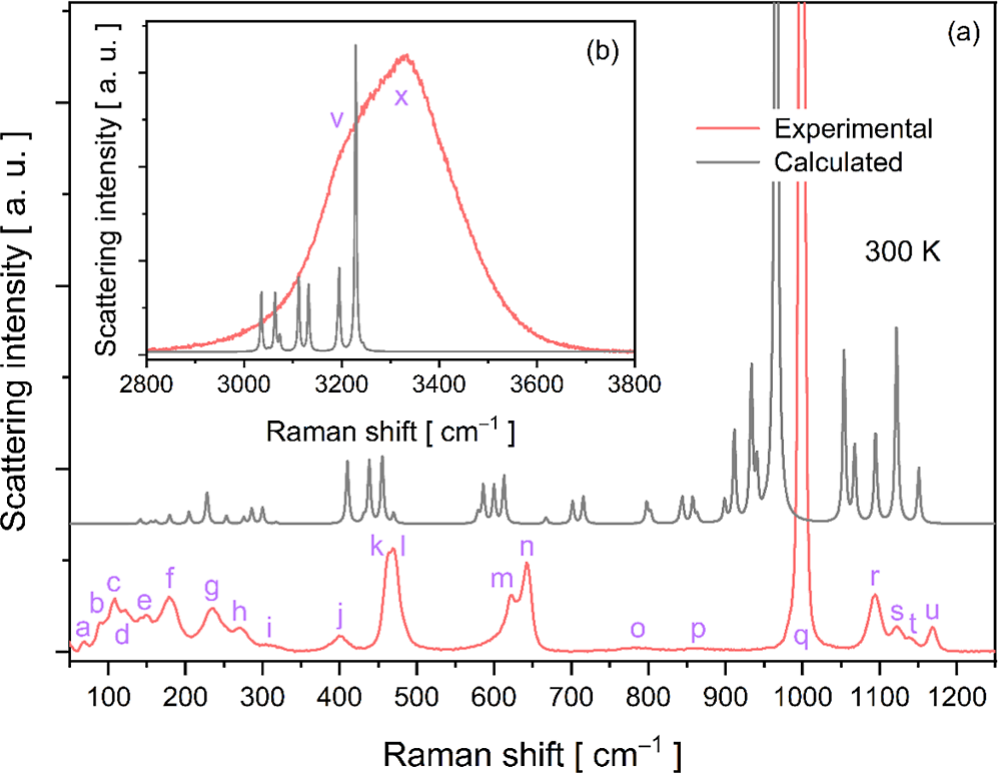
Experimental and calculated Raman spectra of the powdered RbCoSOH Tutton crystal.

In the spectral region of 400 to 1200 cm^–1^, 12 Raman modes (j to u) were recorded, as shown in the spectrum of Figure 7a. Among these modes, characteristic vibrations of the [SO_4_]^2–^ tetrahedra coupled with different deformation modes of H_2_O molecules are observed. A notable example is the most intense band at 999 cm^–1^, assigned to the asymmetric stretching of the [SO_4_]^2–^ tetrahedron with a contribution from the wagging mode of H_2_O units.

Although Tutton salts belong to an isomorphous crystallographic family, most bands exhibit shifts in wavenumber, credited to the influence of mono- and divalent cations in the structure. However, for the sulfate group, only slight shifts are observed compared to other Tutton salts, as the [SO_4_] maintains *C*_1_ site symmetry, as seen in K_2_Ni­(SO_4_)_2_(H_2_O)_6_ and (NH_4_)_2_Fe­(SO_4_)_2_(H_2_O)_6_ crystals.^12,24^

In the 200–310 cm^–1^ range, there are three medium-intensity bands associated with bending modes of the [Co­(H_2_O)_6_]^2+^ complex with contributions from symmetric bending vibrations of the [SO_4_]^2–^ tetrahedra. Below 200 cm^–1^, six bands can be observed corresponding to lattice modes—a spectral region featuring intermolecular vibrations of the entire crystal lattice, involving couplings: (i) translational modes of Rb^+^ cations, (ii) bending/torsion modes of the [Co­(H_2_O)_6_]^2+^ complex, and (iii) bending modes of the [SO_4_]^2–^ tetrahedra. This spectral region exhibits sensitivity to structural changes induced by thermal and pressure variations, making it crucial for identifying phase transition/transformation events in the material.

### 3.5 Thermal Behavior

Figure 8 displays the simultaneous TG-DSC thermograms recorded between 300 and 700 K. At the beginning of the thermogram, the TG curve shows no significant mass loss up to 330.3 K, confirming thermal stability in this range. Beyond this temperature, a considerable mass loss (20.57% of the initial mass (2.21 mg), equivalent to 0.455 mg or 109.04 g/mol) in a single step occurs, corresponding to the release of six H_2_O molecules (theoretical = 108.09 g/mol) coordinated to the cobalt metal center. The endothermic peak at 383.8 K in the DSC curve confirms the phase transition from the hexahydrate to the anhydrous form (Rb_2_Co­(SO_4_)_2_(H_2_O)_6_ → Rb_2_Co­(SO_4_)_2_), with a dehydration enthalpy (Δ*H*) of 301.15 kJ/mol (50.19 kJ per H_2_O molecule). These parameters underscore the potential of the RbCoSOH for low-temperature thermochemical energy storage, owing to its low dehydration temperature and high enthalpy, comparable to the salts (NH_4_)_2_Ni­(SO_4_)_2_(H_2_O)_6_ and (NH_4_)_2_Zn­(SO_4_)_2_(H_2_O)_6_.^58^

**Figure 8:**
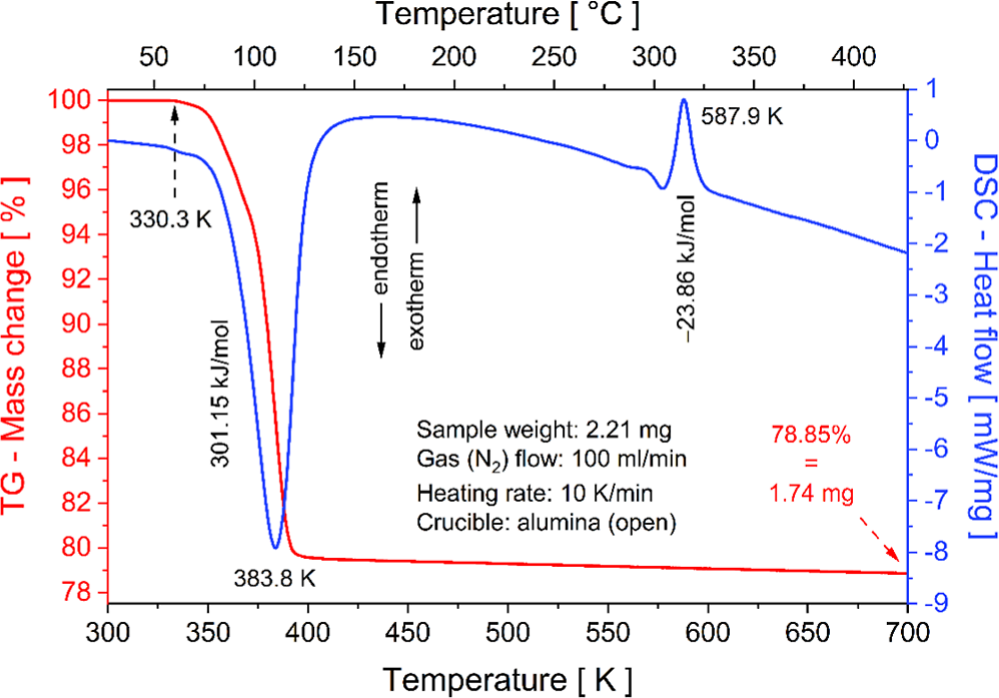
Simultaneous TG-DSC thermograms of RbCoSOH crystal in powder form.

Above 400 K, the salt remains stable in the anhydrous phase up to 700 K (TG curve). However, the DSC curve exhibits an exothermic peak at 587.9 K (Δ*H* = −23.86 kJ/mol) attributed to crystallization. The dehydration of the [Co­(H_2_O)_6_]^2+^ complex induces structural distortions and amorphization due to the breaking of chemical bonds. However, above 570 K, Co^2+^–[SO_4_]^2–^ bonding is favored, promoting the crystallization of a new anhydrous phase (e.g., Rb_2_Co_2_(SO_4_)_3_ or Rb_2_Co_3_(SO_4_)_4_). A similar behavior was observed for (NH_4_K)­Co­(SO_4_)_2_(H_2_O)_6_ Tutton salt, which transits to K_2_Co_2_(SO_4_)_3_ upon dehydration, followed by crystallization.^6^

### 3.6 Optical Response

Figure 9 exhibits the UV-vis-NIR optical absorbance spectrum (unpolarized light) of a RbCoSOH single crystal (unoriented), where the [Co­(H_2_O)_6_]^2+^ complex features the metal in the +2 oxidation state, coordinated by six H_2_O molecules acting as weak-field ligands.^59^ In the UV region (below 400 nm), the well-defined bands at 226, 270, and 292 nm supposedly arise from very high-energy charge-transfer bands and internal transitions within the sulfate anion. It is believed that the most significant source of UV absorption is the ligand-to-metal charge transfer (LMCT) band; a high-energy process where an electron is excited from a nonbonding orbital of a water (H_2_O) ligand to an empty or partially filled *d*-orbital of the central Co^2+^ ion. However, the contribution from intraligand transitions of sulfate anions cannot be ruled out. This case involves exciting an electron from a nonbonding lone pair on one of the oxygen atoms to a higher-energy antibonding orbital (σ*) within the sulfate ion itself (known as *n* → σ* transition). Anyway, the optical gap is defined by the abrupt increase in absorbance at ≈ 300 nm (≈ 4.13 eV). On the other hand, the two bands peaked at 512 and 640 (very low intensity) nm in the vis region, corresponding to the *d*-*d* transitions ^4^T_1g_(^4^ *F*) → ^4^T_1g_(^4^ *P*) and ^4^T_1g_(^4^ *F*) → ^4^A_2g_(^4^ *F*),^16^ respectively, characteristic of Co^2+^ in a slightly distorted octahedral environment.

**Figure 9:**
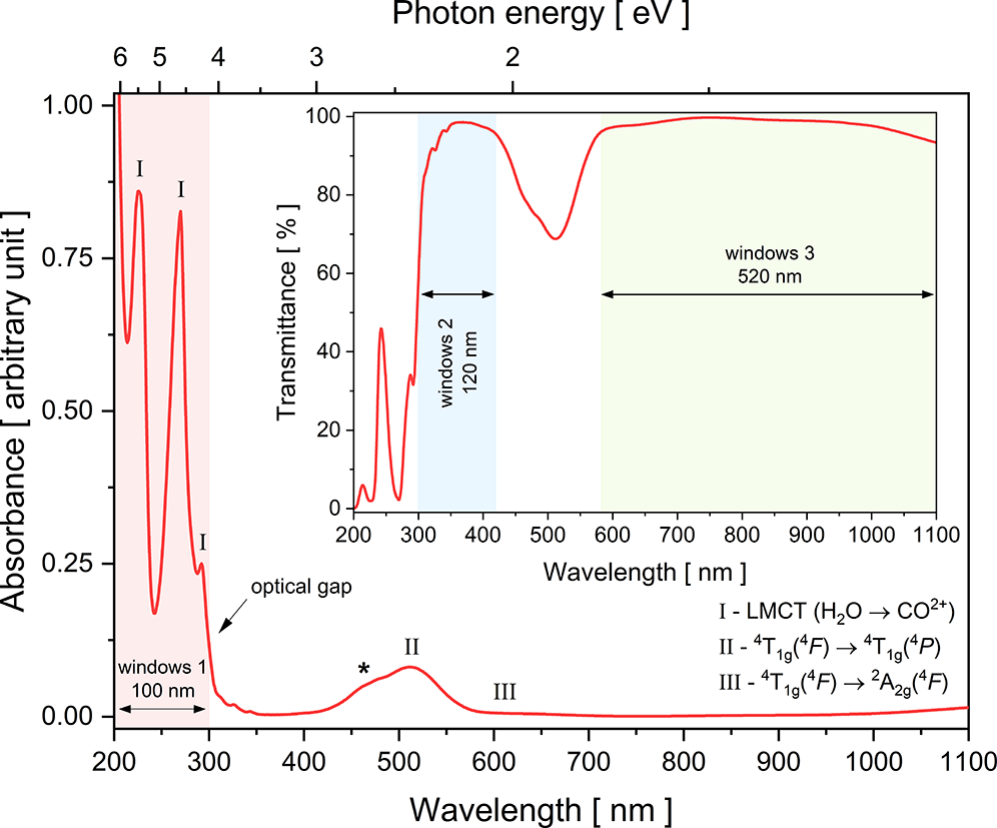
UV-vis-NIR absorbance spectrum of a RbCoSOH single crystal. The symbol * indicates the Jahn-Teller effect observed at around 475 nm. Inset: Corresponding optical transmittance spectrum.

The shoulder at 475 nm (denoted by * in Figure 8), associated with the most intense band, reflects the Jahn-Teller effect, arising from the instability of the degenerate ^4^T_1g_ ground state in the *d* ^7^ configuration (specifically *t*_2g_^5^ *e*_g_^2^) under ideal octahedral symmetry, leading to symmetry breaking that removes degeneracy and causes the observed splitting. Therefore, the only role of the Rb^+^ ion in this crystal is structural; its positive charge balances the negative charge of the sulfate anions, holding the crystal lattice together through electrostatic forces. It does not participate in the UV absorption or vis light. However, the primary way Rb^+^ influences the optical transitions is by modifying the crystal field (or ligand field) around the cobalt ion and consequently altering the transition energy. The *d*-orbitals of the Co, and thus the energy of their *d*-*d* transitions, are extremely sensitive to the precise distance and arrangement of the H_2_O ligands. Even a minimal distortion changes the ligand field strength, which in turn shifts the energy of the optical absorption. This is why different [Co­(H_2_O)_6_]^2+^ complex-based Tutton salts, but with different alkali metals (e.g., K^+^ ^11,15,60^ and [NH_4_]^+^ ^9,16^ ), have slightly different absorption spectra and subtly different shades of color. The different size and charge density of the alkali metal cation changes the crystal structure enough to “tune” the *d-d* transition energies.

Figure 9 inset depicts the optical transmittance spectrum of the same single crystal, showing in another way the three distinct windows: one with high blocking and two with near 100% translucency. The window 1 covers the UV-C (200–300 nm) region, window 2 the UV-B/UV-A/vis (300–420 nm) interval, while window 3 extends the vis-NIR range (580–1100 nm). High absorbance and transmittance levels in specific spectral ranges, i.e., selective optical behavior, enable innovative technologies, including high-sensitivity UV sensors (solar-blind technology) and bandpass filters. Currently, most modern optical filters are developed using multilayer thin films composed of organic and inorganic materials deposited on translucent substrates. In this context, the RbCoSOH in its single-crystal form and its light absorbance/transmittance nature can eliminate the need to deposit optical coatings on a substrate.^17^

It is worth noting that the electronic bandgap (≈ 3.00 eV) differs from the optical bandgap (≈ 4.13 eV), despite the same crystal packing influencing both physical parameters. Typically, the optical bandgap is less than the electronic bandgap. The electronic bandgap is the minimum energy required to create a free electron and a free hole, while the optical bandgap is the minimum energy required for a photon to be absorbed, which establishes an exciton (bound electron–hole pair). The optical bandgap determines the exact wavelengths of light a device can absorb or emit, enabling applications in photovoltaics and LEDs (utilizing wide-bandgap materials) to infrared sensors (utilizing narrow-bandgap materials).^61,62^

However, there are specific scenarios where the measured optical gap can be larger than the fundamental electronic gap. The explanation is based on band filling, i.e., electrons filling the lowest available states in the conduction band (up to the Fermi level). However, the Pauli exclusion principle states that no two electrons can occupy the same state. Because the lowest states in the conduction band are already full, an electron from the valence band can not be excited into them. For a photon to be absorbed, it must have enough energy to promote an electron from the valence band to the first available empty state in the conduction band, which is now at a much higher energy level. This means the energy required for optical absorption is now significantly larger than the intrinsic electronic gap of the material.

The high absorbance in the UV-C region is comparable to that of other Tutton salts reported in the literature and appears to be a signature of sulfated Tutton crystals. For instance, (NH_4_)_2_Fe­(SO_4_)_2_(H_2_O)_6_ exhibits a narrow UV-C light-absorbing window (190–280 nm) followed by a wide transmission window (400–810 nm).^12^ Similarly, the (NH_4_)_2_Mn_0.47_Cu_0.53_(SO_4_)_2_(H_2_O)_6_ crystal have shown a deep light blocking in the UV-C (190–280 nm) and vis/NIR spectral regions (615 to 1000 nm) and high transmittance levels (reaching ≈ 98.5%) in the UV-B/UV-A/vis range (280–615 nm), attributed to the Cu^2+^ and Mn^2+^ coordination environment.^17^ On the other hand, the (NH_4_)_2_Fe_0.11_Ni_0.89_(SO_4_)_2_(H_2_O)_6_ has a selective UV-B filtering capability (280–315 nm).^18^

In RbCoSOH, the inherent Jahn-Teller distortion and the asymmetric arrangement of Co^2+^ octahedra in the monoclinic structure give rise to direction-dependent optical transitions. The arrangement of components in the unit cell follows monoclinic symmetry (low degree of symmetry) and local distortion (Jahn-Teller distortion is the first source of asymmetry). In fact, the general optical behavior is influenced by the local (site) and extended (lattice) anisotropic nature. A photoresponse dependent on the direction of the crystallographic axis offers potential for polarization-sensitive optical filtering. Further analysis of the RbCoSOH crystal should go in this direction, i.e. optical measurements with polarized light.

## 4 Conclusions

This study provided a comprehensive understanding of the Rb_2_Co­(SO_4_)_2_(H_2_O)_6_ Tutton salt through combining experimental techniques and theoretical methods. Single crystals were grown via slow solvent evaporation and found to crystallize in the monoclinic *P*2_1_/*a* space group. The structural integrity and stability of the salt are underpinned by a dense hydrogen-bonding lattice, a conclusion supported by Hirshfeld surface analysis, which revealed a minimal void volume of only 1.41%. Vibrational properties were explored using infrared and Raman spectroscopy, which showed several active optical phonon modes out of the 183 theoretically possible. The assignment of these modes was made using DFT calculations. Thermal analysis showed a single-step, complete dehydration process occurring at ≈approximately 384 K, accompanied by a significant enthalpy change of Δ*H* = 301.15 kJ/mol, indicating promising low-temperature thermochemical energy storage. Optical characterization identified distinct transmittance windows in the UV-B/UV-A/vis and vis-NIR regions (300–420 nm and 580–1100 nm). Furthermore, absorbance bands in the UV-C (200–300 nm) and vis (420–580 nm) spectra were attributed to O^2–^ → Co^2+^ LMCT and intra-atomic Co^2+^ *d*-*d* electronic transitions, respectively. The optical bandgap was estimated to be 3.00 eV. These optical properties indicate a potential use of the crystal in solar-blind UV-C sensors and selective light-filtering devices. In conclusion, this work establishes a robust structure-property relationship for Rb_2_Co­(SO_4_)_2_(H_2_O)_6_, advancing the fundamental science of Tutton salts and demonstrating their feasibility for functional applications. The findings provide a clear directive for future research focused on tuning the thermal and optical properties through strategic substitution of monovalent or divalent cations.

## Supplementary Material



## Notes

Partial density of states contribution by atoms and orbitals; calculated normal modes, irreducible representations, IR and Raman activity modes (PDF)
