# Supplementary material for: Properties and Perspectives of Rb2Co(SO4)2(H2O)6 Tutton Crystal: A Combined Experimental-Theoretical Analysis
Source: ACS Omega. 2025 Oct 13;10(41):49074–86. doi: 10.1021/acsomega.5c07896 (PMC12547550; doi:10.1021/acsomega.5c07896)
Supplement: Supplementary file 1 [file ao5c07896_si_001.pdf]

Supplementary material

Properties and perspectives of $Rb_2Co(SO_4)_2(H_2O)_6$ Tutton crystal: a combined experimental-theoretical analysis

João G. de Oliveira Neto[a,*], Letícia F. Gomes[a], Francisco W. S. de Sousa Junior[a], Djany S. Silva[a], Kamila R. Abreu[a], Luiz F. L. da Silva[b], Luzeli M. da Silva[a], Pedro de F. Façanha Filho[a], Eliana B. Souto[c], Adenilson O. dos Santos[a], and Rossano Lang[d,**]

[a] Center for Social Sciences, Health, and Technology, Federal University of Maranhão UFMA, Imperatriz, MA, 65900-410, Brazil

[b] Criminalistics Institute, Scientific Police of Pará, Marabá, PA, 68507-000, Brazil

[c] UCD School of Chemical and Bioprocess Engineering, University College Dublin, Belfield, Dublin 4, D04 V1W8, Ireland

[d] Institute of Science and Technology, Federal University of São Paulo UNIFESP, São José dos Campos, SP, 12231-280, Brazil

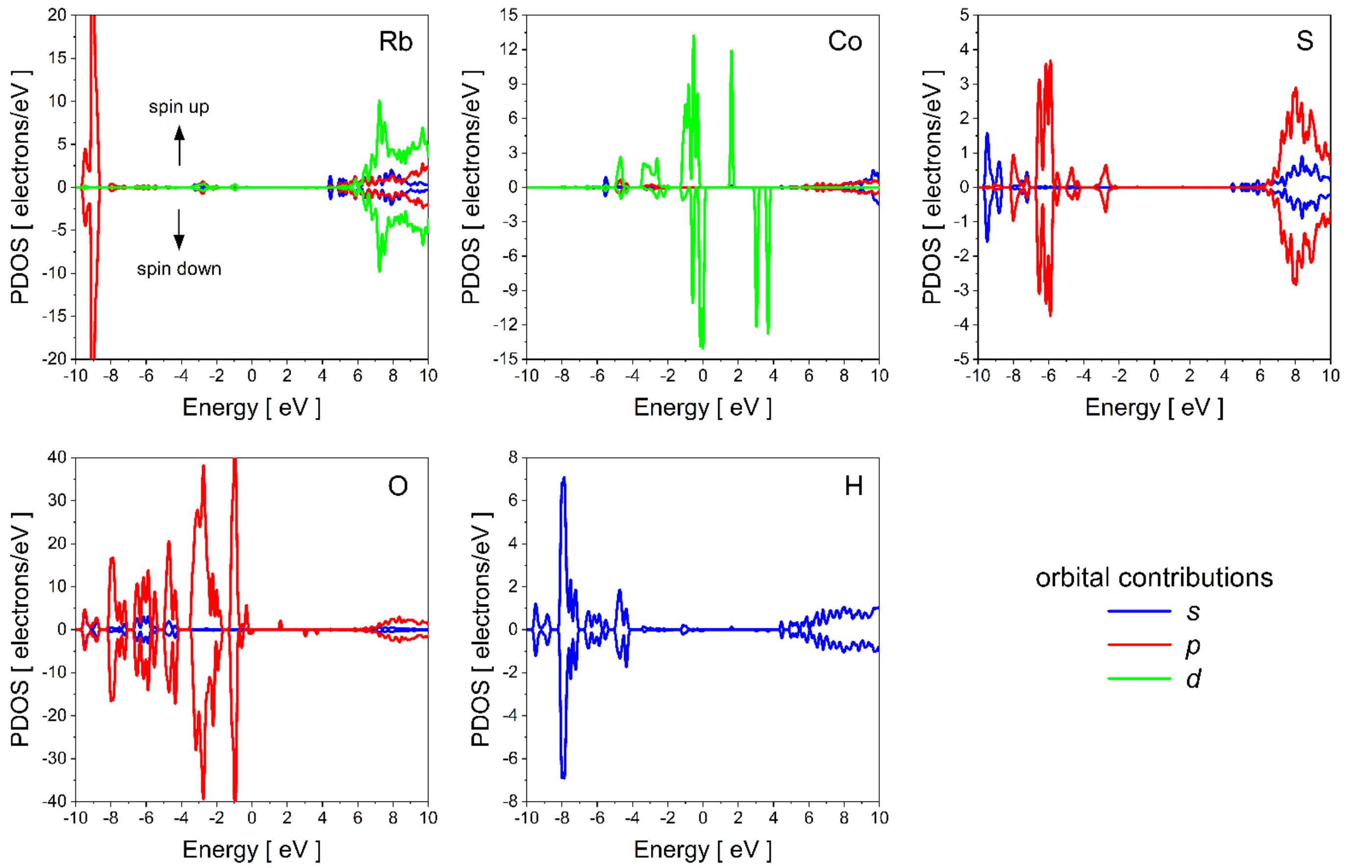

Figure S1. Partial density of states contribution of $Rb_2Co(SO_4)_2(H_2O)_6$ crystal by atoms and orbitals.

Table S1. Calculated normal modes, irreducible representations (Irrep.), IR and Raman activity modes for the $Rb_2Co(SO_4)_2(H_2O)_6$ Tutton salt.

N°	ω [cm^{-1}]	Irrep	IR	Raman
1	50	Ag	N	Y
2	55	Au	Y	N
3	60	Au	Y	N
4	61	Ag	N	Y
5	68	Bg	N	Y
6	75	Bu	Y	N
7	76	Bg	N	Y
8	77	Au	Y	N
9	78	Ag	N	Y
10	79	Bu	Y	N
11	80	Bg	N	Y
12	81	Au	Y	N
13	82	Bu	Y	N
14	86	Bg	N	Y
15	87	Ag	N	Y
16	88	Bu	Y	N
17	90	Au	Y	N
18	95	Au	Y	N
19	96	Ag	N	Y
20	101	Bg	N	Y
21	104	Ag	N	Y
22	107	Au	Y	N
23	109	Bu	Y	N
24	114	Bg	N	Y
25	116	Bu	Y	N
26	118	Au	Y	N
27	119	Bu	Y	N
28	123	Ag	N	Y
29	127	Bg	N	Y
30	128	Bu	Y	N
31	133	Au	Y	N
32	136	Bg	N	Y
33	141	Ag	N	Y
34	145	Bg	N	Y
35	145	Au	Y	N
36	146	Au	Y	N
37	153	Bu	Y	N
38	155	Ag	N	Y
39	159	Bu	Y	N
40	161	Ag	N	Y
41	162	Bg	N	Y
42	171	Bu	Y	N
43	174	Bg	N	Y
44	175	Au	Y	N
42	171	Bu	Y	N
43	174	Bg	N	Y
44	175	Au	Y	N
42	171	Bu	Y	N
43	174	Bg	N	Y
44	175	Au	Y	N
45	179	Ag	N	Y
46	189	Au	Y	N
47	190	Bu	Y	N
48	195	Bg	N	Y

49	202	Au	Y	N
50	204	Bu	Y	N
51	205	Ag	N	Y
52	227	Bg	N	Y
53	228	Ag	N	Y
54	230	Au	Y	N
55	231	Bu	Y	N
56	253	Ag	N	Y
57	254	Bu	Y	N
58	274	Au	Y	N
59	276	Bg	N	Y
60	278	Bu	Y	N
61	280	Au	Y	N
62	286	Ag	N	Y
63	289	Bg	N	Y
64	300	Ag	N	Y
65	308	Bg	N	Y
66	318	Ag	N	Y
67	319	Bg	N	Y
68	321	Au	Y	N
69	328	Bu	Y	N
70	336	Bu	Y	N
71	338	Au	Y	N
72	410	Ag	N	Y
73	411	Bg	N	Y
74	424	Bu	Y	N
75	427	Au	Y	N
76	431	Bg	N	Y
77	434	Au	Y	N
78	438	Ag	N	Y
79	441	Bu	Y	N
80	455	Ag	N	Y
81	459	Bu	Y	N
82	469	Au	Y	N
83	470	Bg	N	Y
84	576	Bu	Y	N
85	579	Ag	N	Y
86	581	Au	Y	N
87	586	Bg	N	Y
88	596	Au	Y	N
89	597	Bu	Y	N
90	599	Bg	N	Y
91	600	Ag	N	Y
92	607	Au	Y	N
93	609	Ag	N	Y
94	611	Bg	N	Y
95	613	Ag	N	Y
96	658	Bu	Y	N
97	661	Au	Y	N
98	666	Bu	Y	N
99	668	Ag	N	Y
100	682	Au	Y	N
101	693	Bu	Y	N
102	702	Ag	N	Y
103	706	Au	Y	N
104	708	Bg	N	Y
105	714	Bg	N	Y
106	715	Ag	N	Y

107	716	Bu	Y	N
108	797	Bu	Y	N
109	798	Ag	N	Y
110	803	Bg	N	Y
111	805	Au	Y	N
112	841	Au	Y	N
113	841	Bg	N	Y
114	844	Ag	N	Y
115	845	Bu	Y	N
116	857	Ag	N	Y
117	861	Au	Y	N
118	863	Bg	N	Y
119	871	Bu	Y	N
120	899	Bg	N	Y
121	905	Au	Y	N
122	911	Ag	N	Y
123	912	Bu	Y	N
124	933	Ag	N	Y
125	937	Bu	Y	N
126	940	Bg	N	Y
127	941	Ag	N	Y
128	942	Ag	Y	N
129	944	Bu	Y	N
130	945	Au	Y	N
131	951	Bu	Y	N
132	963	Bu	Y	N
133	964	Au	Y	N
134	965	Au	N	Y
135	969	Bg	N	Y
136	1053	Ag	N	Y
137	1054	Bg	N	Y
138	1055	Au	Y	N
139	1061	Bu	Y	N
140	1068	Ag	N	Y
141	1080	Bu	Y	N
142	1086	Au	Y	N
143	1095	Bg	N	Y
144	1119	Bu	Y	N
145	1122	Ag	N	Y
146	1131	Au	Y	N
147	1150	Bg	N	Y
148	1551	Bg	N	Y
149	1558	Au	Y	N
150	1559	Bu	Y	N
151	1562	Bg	N	Y
152	1586	Au	Y	N
153	1587	Bg	N	Y
154	1590	Bu	Y	N
155	1594	Ag	N	Y
156	1597	Ag	N	Y
157	1598	Bu	Y	N
158	1602	Au	Y	N
159	1605	Bu	Y	N
160	3034	Au	Y	N
161	3035	Ag	N	Y
162	3040	Bu	Y	N
163	3050	Bg	N	Y
164	3054	Bu	Y	N

165	3064	Ag	N	Y
166	3066	Au	Y	N
167	3073	Bg	N	Y
168	3112	Ag	N	Y
169	3113	Bg	N	Y
170	3115	Au	Y	N
171	3117	Bu	Y	N
172	3132	Ag	N	Y
173	3137	Bu	Y	N
174	3139	Au	Y	N
175	3191	Bg	N	Y
176	3193	Bu	Y	N
177	3195	Ag	N	Y
178	3198	Bg	N	Y
179	3202	Ag	Y	N
180	3213	Bu	Y	N
181	3224	Au	Y	N
182	3225	Au	Y	N
183	3244	Bg	N	Y
